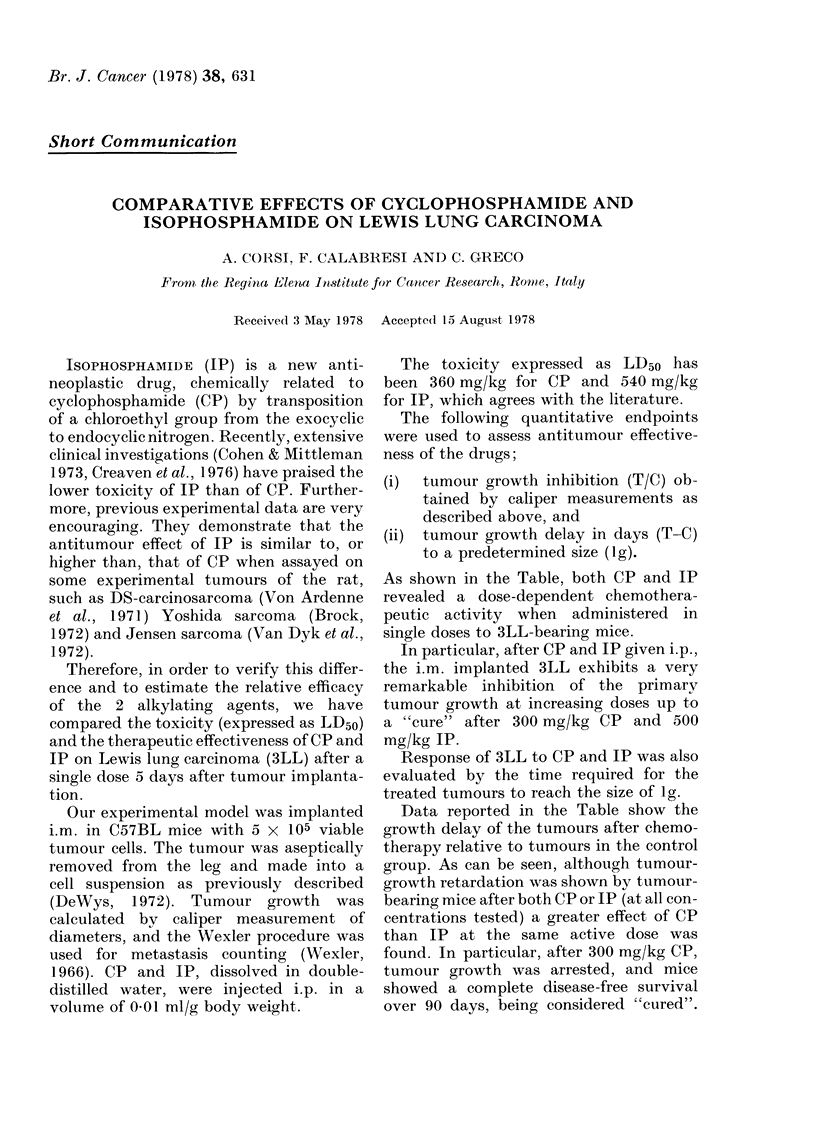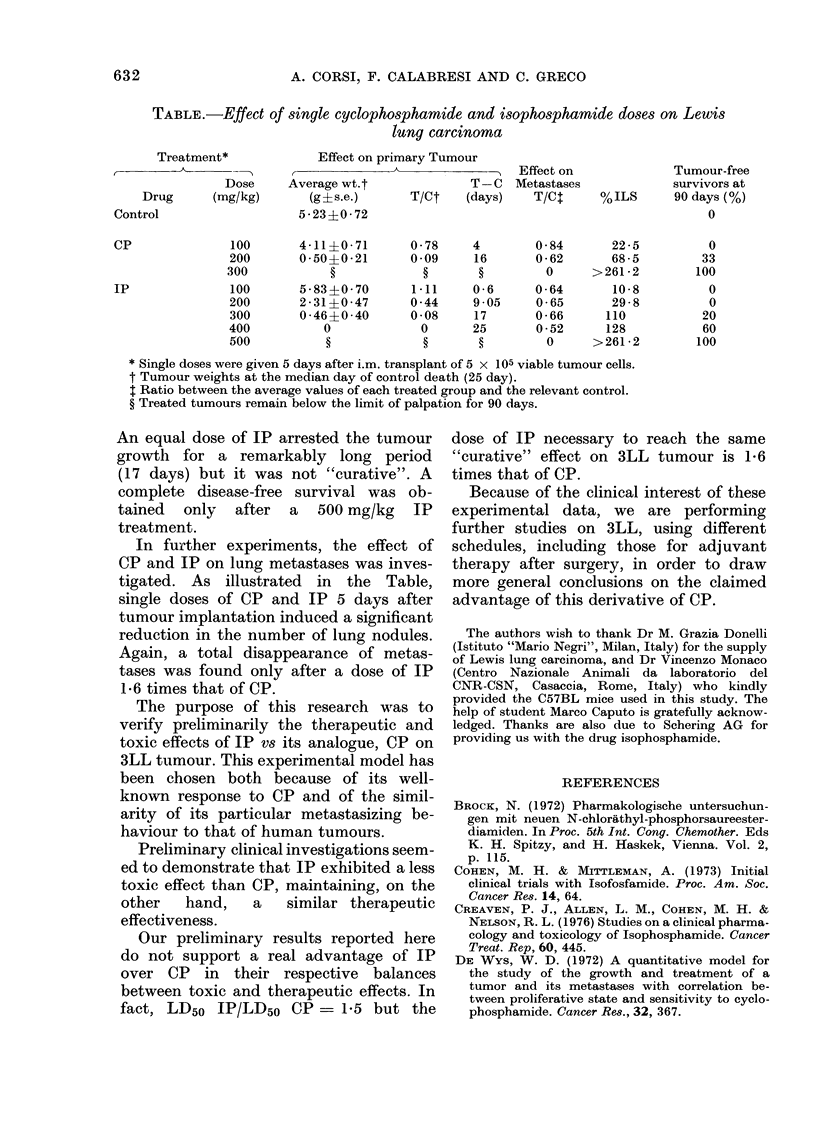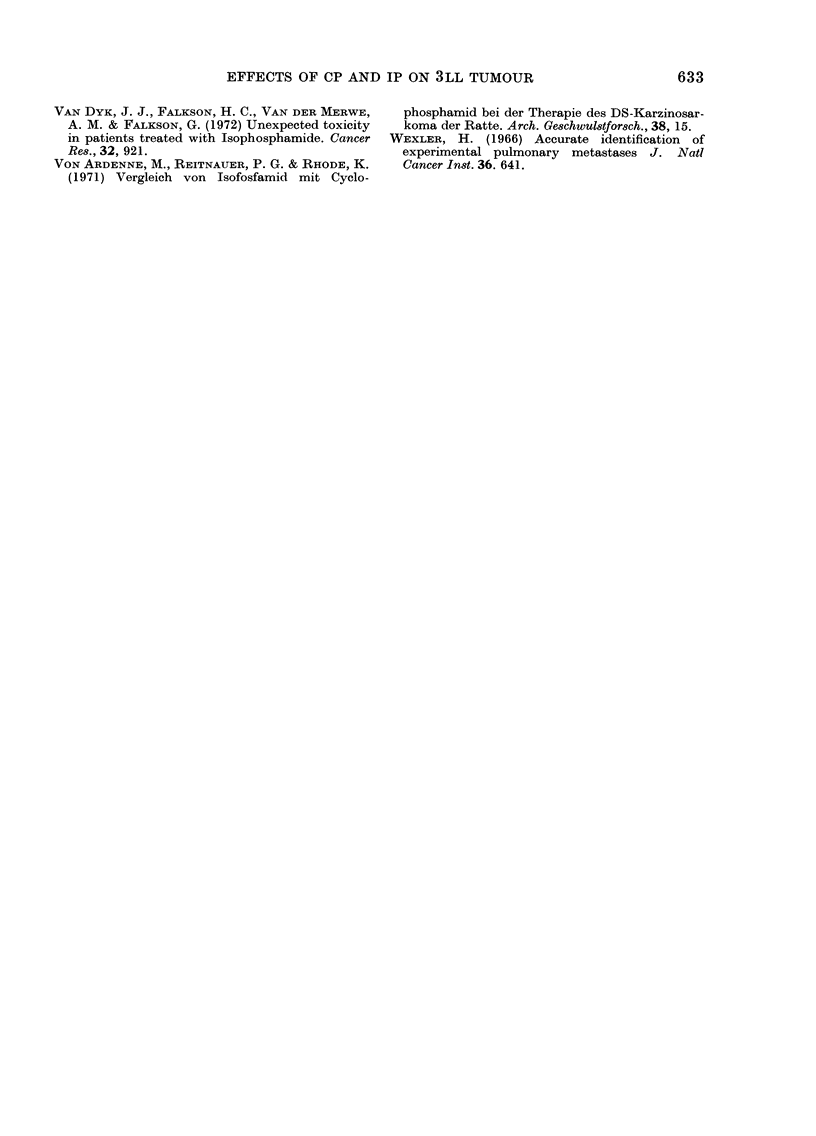# Comparative effects of cyclophosphamide and isophosphamide on Lewis lung carcinoma.

**DOI:** 10.1038/bjc.1978.260

**Published:** 1978-11

**Authors:** A. Corsi, F. Calabresi, C. Greco


*Br. J. Cancer* (1978) **38**, 631

***Short Communication***

### COMPARATIVE EFFECTS OF CYCLOPHOSPHAMIDE AND ISOPHOSPHAMIDE ON LEWIS LUNG CARCINOMA

A. CORSI, F. CALABRESI AND C. GRECO

*From the Regina Elena Institute for Cancer Research, Rome, Italy*

Received 3 May 1978 Accepted 15 August 1978

ISOPHOSPHAMIDE (IP) is a new antineoplastic drug, chemically related to cyclophosphamide (CP) by transposition of a chloroethyl group from the exocyclic to endocyclic nitrogen. Recently, extensive clinical investigations (Cohen & Mittleman 1973, Creaven *et al.*, 1976) have praised the lower toxicity of IP than of CP. Furthermore, previous experimental data are very encouraging. They demonstrate that the antitumour effect of IP is similar to, or higher than, that of CP when assayed on some experimental tumours of the rat, such as DS-carcinosarcoma (Von Ardenne *et al.*, 1971) Yoshida sarcoma (Brock, 1972) and Jensen sarcoma (Van Dyk *et al.*, 1972).

Therefore, in order to verify this difference and to estimate the relative efficacy of the 2 alkylating agents, we have compared the toxicity (expressed as $LD_{50}$) and the therapeutic effectiveness of CP and IP on Lewis lung carcinoma (3LL) after a single dose 5 days after tumour implantation.

Our experimental model was implanted i.m. in C57BL mice with $5 \times 10^5$ viable tumour cells. The tumour was aseptically removed from the leg and made into a cell suspension as previously described (DeWys, 1972). Tumour growth was calculated by caliper measurement of diameters, and the Wexler procedure was used for metastasis counting (Wexler, 1966). CP and IP, dissolved in double-distilled water, were injected i.p. in a volume of 0·01 ml/g body weight.

The toxicity expressed as $LD_{50}$ has been 360 mg/kg for CP and 540 mg/kg for IP, which agrees with the literature.

The following quantitative endpoints were used to assess antitumour effectiveness of the drugs;

(i) tumour growth inhibition (T/C) obtained by caliper measurements as described above, and
(ii) tumour growth delay in days (T–C) to a predetermined size (1g).

As shown in the Table, both CP and IP revealed a dose-dependent chemotherapeutic activity when administered in single doses to 3LL-bearing mice.

In particular, after CP and IP given i.p., the i.m. implanted 3LL exhibits a very remarkable inhibition of the primary tumour growth at increasing doses up to a "cure" after 300 mg/kg CP and 500 mg/kg IP.

Response of 3LL to CP and IP was also evaluated by the time required for the treated tumours to reach the size of 1g.

Data reported in the Table show the growth delay of the tumours after chemotherapy relative to tumours in the control group. As can be seen, although tumour-growth retardation was shown by tumour-bearing mice after both CP or IP (at all concentrations tested) a greater effect of CP than IP at the same active dose was found. In particular, after 300 mg/kg CP, tumour growth was arrested, and mice showed a complete disease-free survival over 90 days, being considered "cured".

TABLE.—*Effect of single cyclophosphamide and isophosphamide doses on Lewis lung carcinoma*

| Treatment* | | Effect on primary Tumour | | | Effect on Metastases | | Tumour-free survivors at |
|---|---|---|---|---|---|---|---|
| Drug | Dose (mg/kg) | Average wt.† (g±s.e.) | T/C† | T−C (days) | T/C‡ | %ILS | 90 days (%) |
| Control | | 5·23±0·72 | | | | | 0 |
| CP | 100 | 4·11±0·71 | 0·78 | 4 | 0·84 | 22·5 | 0 |
| | 200 | 0·50±0·21 | 0·09 | 16 | 0·62 | 68·5 | 33 |
| | 300 | § | § | § | 0 | >261·2 | 100 |
| IP | 100 | 5·83±0·70 | 1·11 | 0·6 | 0·64 | 10·8 | 0 |
| | 200 | 2·31±0·47 | 0·44 | 9·05 | 0·65 | 29·8 | 0 |
| | 300 | 0·46±0·40 | 0·08 | 17 | 0·66 | 110 | 20 |
| | 400 | 0 | 0 | 25 | 0·52 | 128 | 60 |
| | 500 | § | § | § | 0 | >261·2 | 100 |

* Single doses were given 5 days after i.m. transplant of $5 \times 10^5$ viable tumour cells.
† Tumour weights at the median day of control death (25 day).
‡ Ratio between the average values of each treated group and the relevant control.
§ Treated tumours remain below the limit of palpation for 90 days.

An equal dose of IP arrested the tumour growth for a remarkably long period (17 days) but it was not "curative". A complete disease-free survival was obtained only after a 500 mg/kg IP treatment.

In further experiments, the effect of CP and IP on lung metastases was investigated. As illustrated in the Table, single doses of CP and IP 5 days after tumour implantation induced a significant reduction in the number of lung nodules. Again, a total disappearance of metastases was found only after a dose of IP 1·6 times that of CP.

The purpose of this research was to verify preliminarily the therapeutic and toxic effects of IP *vs* its analogue, CP on 3LL tumour. This experimental model has been chosen both because of its well-known response to CP and of the similarity of its particular metastasizing behaviour to that of human tumours.

Preliminary clinical investigations seemed to demonstrate that IP exhibited a less toxic effect than CP, maintaining, on the other hand, a similar therapeutic effectiveness.

Our preliminary results reported here do not support a real advantage of IP over CP in their respective balances between toxic and therapeutic effects. In fact, $LD_{50}$ IP/$LD_{50}$ CP = 1·5 but the dose of IP necessary to reach the same "curative" effect on 3LL tumour is 1·6 times that of CP.

Because of the clinical interest of these experimental data, we are performing further studies on 3LL, using different schedules, including those for adjuvant therapy after surgery, in order to draw more general conclusions on the claimed advantage of this derivative of CP.

The authors wish to thank Dr M. Grazia Donelli (Istituto "Mario Negri", Milan, Italy) for the supply of Lewis lung carcinoma, and Dr Vincenzo Monaco (Centro Nazionale Animali da laboratorio del CNR-CSN, Casaccia, Rome, Italy) who kindly provided the C57BL mice used in this study. The help of student Marco Caputo is gratefully acknowledged. Thanks are also due to Schering AG for providing us with the drug isophosphamide.

### REFERENCES

BROCK, N. (1972) Pharmakologische untersuchungen mit neuen N-chloräthyl-phosphorsaureester-diamiden. In *Proc. 5th Int. Cong. Chemother.* Eds K. H. Spitzy, and H. Haskek, Vienna. Vol. 2, p. 115.

COHEN, M. H. & MITTLEMAN, A. (1973) Initial clinical trials with Isofosfamide. *Proc. Am. Soc. Cancer Res.* **14**, 64.

CREAVEN, P. J., ALLEN, L. M., COHEN, M. H. & NELSON, R. L. (1976) Studies on a clinical pharmacology and toxicology of Isophosphamide. *Cancer Treat. Rep*, **60**, 445.

DE WYS, W. D. (1972) A quantitative model for the study of the growth and treatment of a tumor and its metastases with correlation between proliferative state and sensitivity to cyclophosphamide. *Cancer Res.*, **32**, 367.

Van Dyk, J. J., Falkson, H. C., Van der Merwe, A. M. & Falkson, G. (1972) Unexpected toxicity in patients treated with Isophosphamide. *Cancer Res.*, **32**, 921.

Von Ardenne, M., Reitnauer, P. G. & Rhode, K. (1971) Vergleich von Isofosfamid mit Cyclophosphamid bei der Therapie des DS-Karzinosarkoma der Ratte. *Arch. Geschwulstforsch.*, **38**, 15.

Wexler, H. (1966) Accurate identification of experimental pulmonary metastases *J. Natl Cancer Inst.* **36**. 641.